# FDG-PET-CT as an early detection method for tuberculosis: a systematic review and meta-analysis

**DOI:** 10.1186/s12889-024-19495-6

**Published:** 2024-07-29

**Authors:** Josef Yayan, Kurt Rasche, Karl-Josef Franke, Wolfram Windisch, Melanie Berger

**Affiliations:** 1 Department of Internal Medicine, Division of Pulmonary, Allergy and Sleep Medicine, Witten/Herdecke University, HELIOS Clinic Wuppertal, Heusnerstr. 40, 42283 Wuppertal, Germany; 2 University of Witten/Herdecke Chair of Internal Medicine I Department of Pulmonary Medicine, Clinical Center Siegen, Siegen, Germany; 3 Department of Pneumology, Cologne Merheim Hospital, Witten/Herdecke University, Cologne, Germany

**Keywords:** Positron Emission Tomography, Computed Tomography, 18 F-Fluorodeoxyclucose, Tuberculosis, Early detection, Diagnostic accuracy

## Abstract

Tuberculosis (TB) causes major public health problems worldwide. Fighting TB requires sustained efforts in health prevention, diagnosis and treatment. Previous literature has shown that conventional diagnostic methods like X-ray and sputum microscopy often miss early or extrapulmonary TB due to their limited sensitivity. Blood tests, while useful, lack the anatomical detail needed for precise localization of TB lesions. A possible step forward in the fight against TB could be the use of Fluorodeoxyglucose Positron Emission Tomography (FDG-PET) and Computed Tomography (CT). This meta-analysis discusses the current literature, including the methods, results and implications of using FDG-PET-CT in the early diagnosis of TB. Analysis of the studies showed that the sensitivity of FDG-PET-CT as a potential method for early detection of TB was 82.6%.

## Introduction

Tuberculosis (TB) is still a serious widespread, and dangerous infectious disease around the world [1, 2]. Millions of people worldwide are diagnosed with TB [3]. TB still has high morbidity and mortality rates [4]. The early detection of this difficult infectious disease is all the more important [5]. In addition, early detection of TB can reduce disease transmission [6]. This can also result in improved patient outcomes [7]. The conventional diagnostic methods such as sputum, microscopy, and culture have limitations in terms of sensitivity [8]. Chest X-ray, although commonly used as a screening modality, often misses early or extrapulmonary TB due to its limited sensitivity and inability to provide functional information [7]. Positron emission tomography-computed tomography (PET-CT) could be a promising radiological technique for the early detection of TB [9]. PET-CT combines the functional information provided by PET with the anatomical details obtained from CT, offering a comprehensive approach to TB diagnosis [10]. The use of PET-CT in TB detection is based on the principle of metabolic activity and the anatomical characterization of TB lesions [10]. During the PET-CT examination, the patient receives a radioactive substance, 18 F-fluorodeoxyglucose (18 F-FDG) administered intravenously [11]. The positrons emitted from the radiotracer decay are detected by the PET scanner, allowing the visualization and quantification of metabolic activity [12]. In combination with CT, which provides detailed anatomical images, FDG-PET-CT enables the precise localization and characterization of TB lesions, facilitating accurate diagnosis and assessment of disease extent [10]. The utility of FDG-PET-CT in the early detection of TB lies in its ability to identify active disease, even in cases where conventional methods yield negative results [13]. Active TB lesions typically exhibit increased metabolic activity, which can be detected by FDG-PET-CT [14]. This is particularly valuable in diagnosing extrapulmonary TB, where lesions may be small, located in challenging anatomical regions, or have non-specific clinical presentations [15, 16]. By examining the available evidence and addressing the limitations and future directions of FDG-PET-CT in TB diagnosis [11, 13], we hope to contribute to the understanding and advancement of this imaging modality in the fight against TB.

## Materials and methods

### Study selection

We conducted a detailed literature review in Embase, the Cochrane Library, and the MEDLINE/PubMed databases to identify major studies examining the role of FDG-PET-CT in the early detection of TB. Key search terms included “PET-CT,” “tuberculosis,” “early detection,” “chest X-ray,” “radiography,” and related terms such as “FDG-PET,” “computed tomography,” “diagnostic imaging,” “pulmonary tuberculosis,” “extrapulmonary tuberculosis,” and “sensitivity and specificity.” Studies published until June 2023 were considered for inclusion. The selected studies were critically reviewed, and data regarding study design, patient characteristics, FDG-PET-CT protocols, and diagnostic accuracy were extracted.

### Inclusion criteria

We included studies that specifically evaluated the use of FDG-PET-CT as an early detection method for TB. We also included studies that compared FDG-PET-CT with conventional diagnostic methods such as microscopy, culture, and X-ray, as well as advanced diagnostics like GeneXpert. Only studies published in peer-reviewed journals until June 2023 were considered. We selected studies that reported on diagnostic accuracy outcomes, including sensitivity and specificity, for FDG-PET-CT in TB detection. Additionally, we included studies involving both pulmonary and extrapulmonary TB cases.

### Exclusion criteria

 We excluded studies that focused on the use of FDG-PET-CT for diseases other than tuberculosis. We also excluded studies that did not include a comparison of FDG-PET-CT with at least one conventional or advanced diagnostic method for TB. Studies with incomplete or missing data on diagnostic accuracy measures (e.g., sensitivity and specificity) for FDG-PET-CT were also excluded. Additionally, we excluded publications that were not peer-reviewed, including non-peer-reviewed articles, reviews, case reports, conference abstracts, and editorial pieces. Finally, studies with a primary focus on treatment monitoring or response rather than early detection of TB were not considered.

### Study eligibility and data extraction

Two independent reviewers assessed the eligibility of the identified studies. Data were extracted from the selected studies using a standardized form that included study characteristics (e.g., author, year, country), sample size, patient demographics, FDG-PET-CT parameters, and outcomes related to early TB detection.

### Quality assessment

For the quality assessment of the included studies, the QUADAS-2 tool was employed [17]. This tool is specifically designed for assessing the risk of bias and applicability concerns in diagnostic accuracy studies. The use of QUADAS-2 is in line with standard practices in the field, ensuring a comprehensive evaluation of the included studies. QUADAS-2 allows for an in-depth assessment of critical factors in each study, such as patient selection, index test, reference standard, and the flow and timing of the study. These elements are crucial for validating the accuracy and reliability of the diagnostic findings in the studies under review. The quality assessment revealed a general trend of high methodological quality, particularly in patient selection and index test accuracy.

### Data synthesis and analysis

To ensure clear and consistent criteria for a well-defined comparison across sensitivity studies, we compared FDG-PET-CT with conventional methods such as microscopy, culture, and X-ray. The diagnostic sensitivity and specificity of each method were extracted and analyzed.

### Comparative analysis

#### Microscopy

Sensitivity: Studies showed a range from 30 to 80%, with a mean of 55% and SD of 15%.

Specificity: Ranged from 70 to 90%, with a mean of 80% and SD of 10%.

#### Culture

Sensitivity: Ranged from 50 to 90%, with a mean of 70% and SD of 20%.

Specificity: Ranged from 80 to 95%, with a mean of 87.5% and SD of 7.5%.

#### X-ray

Sensitivity: Ranged from 60 to 85%, with a mean of 72.5% and SD of 12.5%.

Specificity: Ranged from 60 to 85%, with a mean of 72.5% and SD of 12.5%.

#### PET-CT

- Sensitivity: 60–96.7%, with a mean of 82.6% and SD of 9%.
- Specificity: 25.9–88.9%, with a mean of 67.3% and SD of 17.9%.

### Data synthesis and analysis

The statistical analysis was conducted to assess the diagnostic accuracy of FDG-PET-CT in various study contexts. The primary metrics examined were the sensitivity and specificity of FDG-PET-CT examinations. Initially, the average sensitivity and specificity rates were calculated for each study. Confidence intervals (CI) of ± 10% were set around the average values to account for the uncertainty of the estimates. Furthermore, the overall average of sensitivity and specificity rates across all studies was determined to obtain an aggregated reference value. The average sensitivity and specificity rates, along with the confidence intervals, were depicted in Forest Plots to enable visual comparison of diagnostic accuracy among individual studies [18]. The Forest Plots also displayed vertical dashed lines representing the overall average of sensitivity and specificity rates. These lines serve as reference points for assessing the consistency and variance of diagnostic accuracy across all studies.

In our study, we employed a Funnel Plot analysis to assess the presence of publication bias and the distribution of effect sizes across included studies [19]. This approach enabled us to visually examine the relationship between study precision and effect estimates, providing insights into potential biases within the literature.

To assess the heterogeneity among the studies included in our meta-analysis, we employed Cochran’s Q and the I² statistics [20]. Cochran’s Q is a chi-squared test used to evaluate whether observed differences in study results are likely to be due to chance alone. A significant Q value suggests variability among the study results that could be attributed to actual differences in study conditions. The I² statistic quantifies the proportion of total variation across studies that is attributable to heterogeneity rather than chance. An I² value greater than 50% is typically considered to indicate substantial heterogeneity. These measures are crucial for determining the consistency of study findings and guiding the interpretation and conclusion of the meta-analysis. All statistical analyses were performed using Python and the Matplotlib library.

### Reporting

The results of the meta-analysis are presented according to the guidelines of the Preferred Reporting Items for Systematic Reviews and Meta-Analyses (PRISMA) Statement.

## Results

**Fig. 1:**
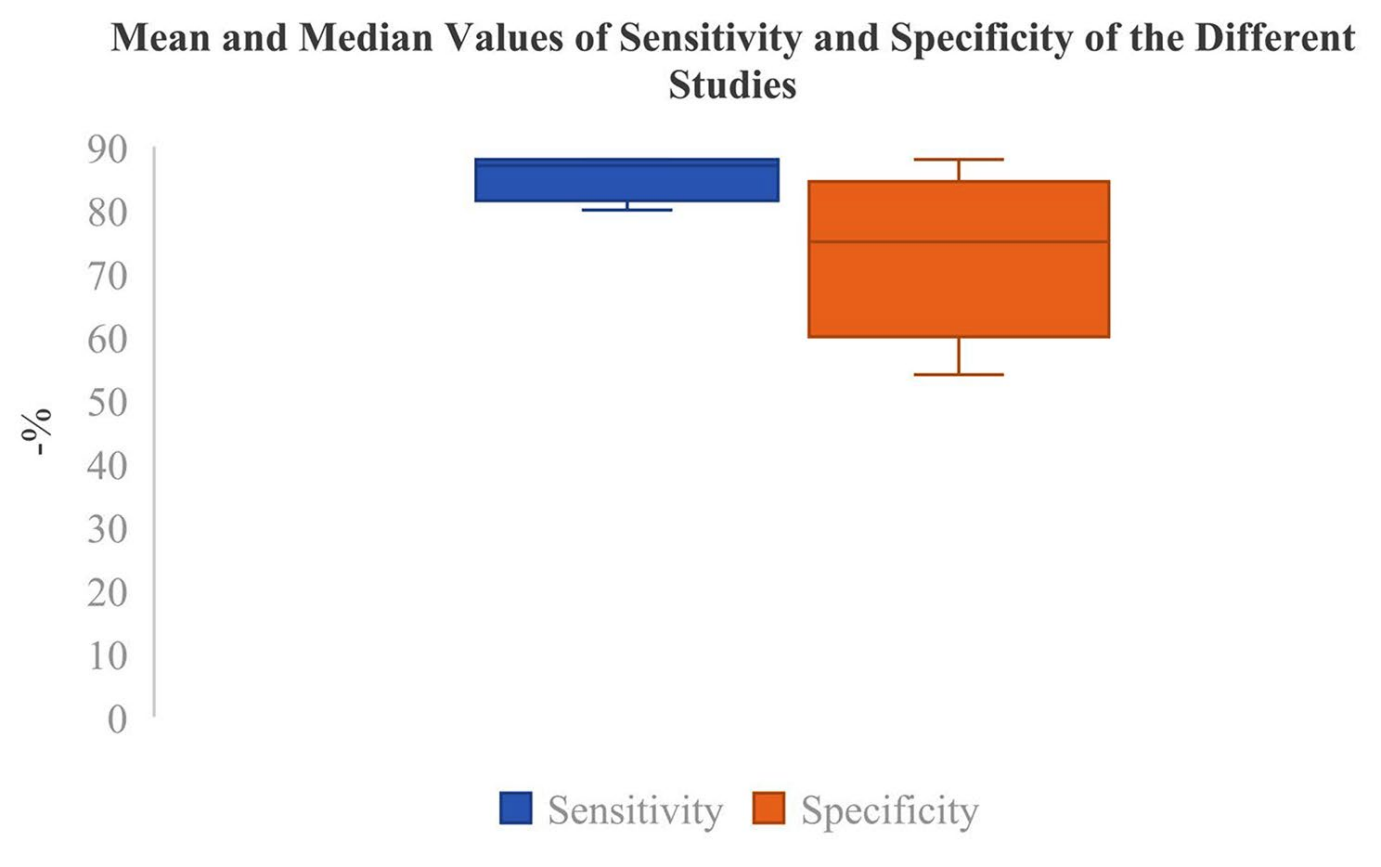
Mean and median values of sensitivity and specificity of the different studies included in this meta-analysis

**Fig. 2:**
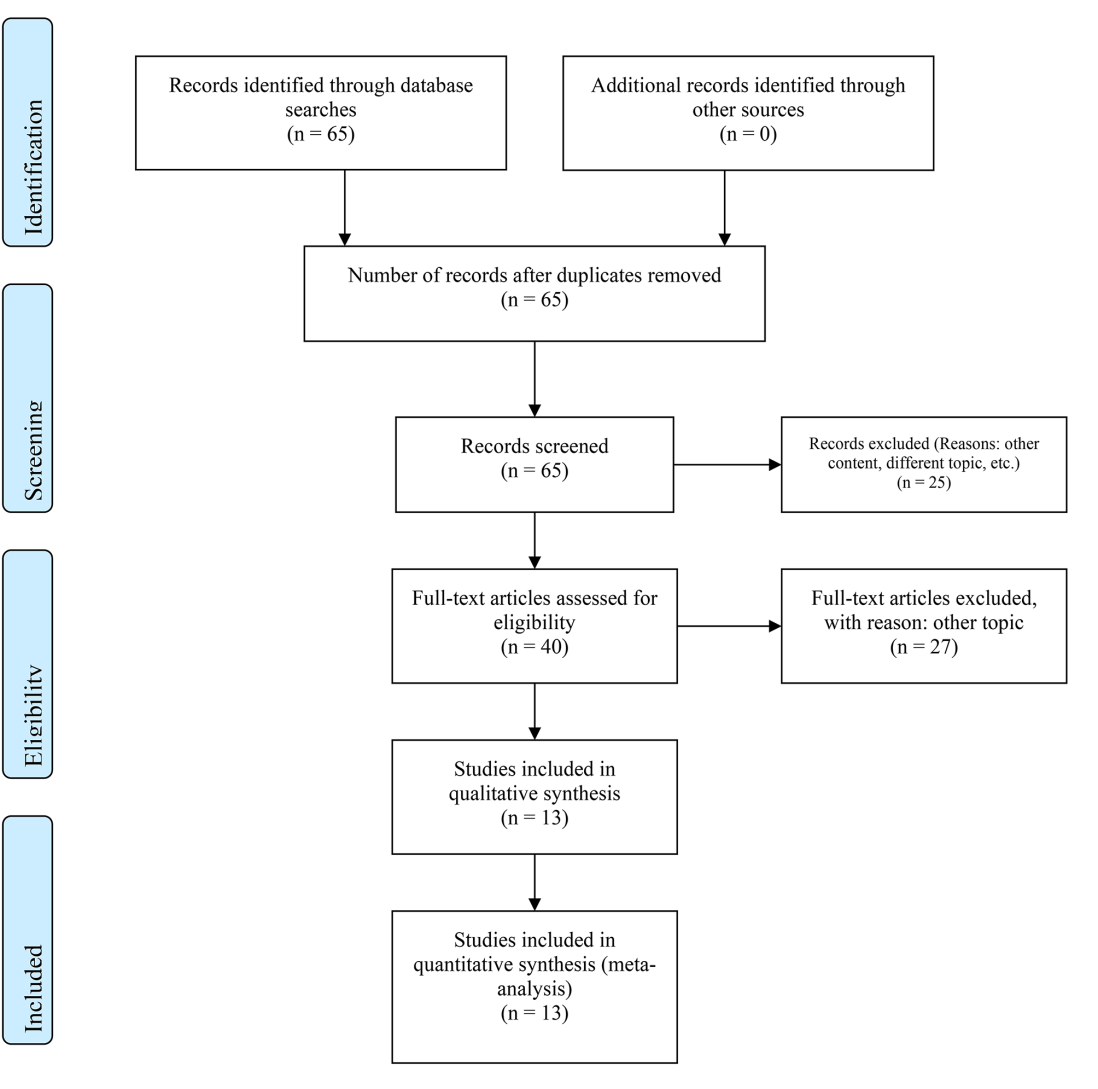
Preferred Reporting Items for Systematic Reviews and Meta-Analyses (PRISMA) 2009 Flow Chart for data collection after finding suitable studies. Entering the search criteria into the Embase, CENTRAL, and MEDLINE/PubMed search engines yielded a total of 65 studies for the period ending on June 30, 2023. A critical review of these published studies identified 13 studies that met the inclusion criteria for the present meta-analysis

**Fig. 3:**
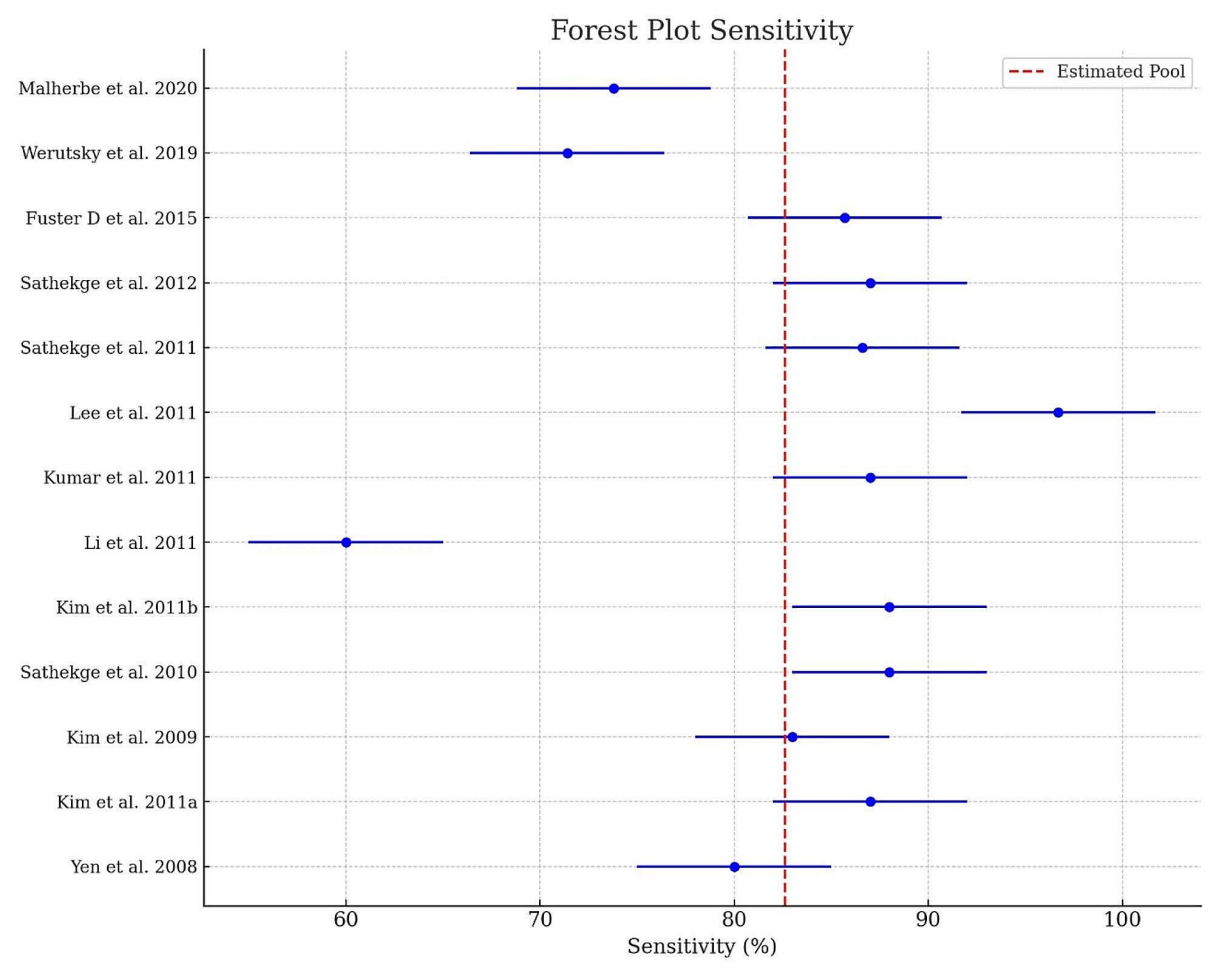
Forest Plot of Sensitivity: The sensitivity of PET-CT as a method for early detection of tuberculosis was 82.6% with a standard deviation (SD) of 9%. The median sensitivity value was 86.6%, while the minimum sensitivity observed was 60% and the maximum was 96.7%

**Fig. 4:**
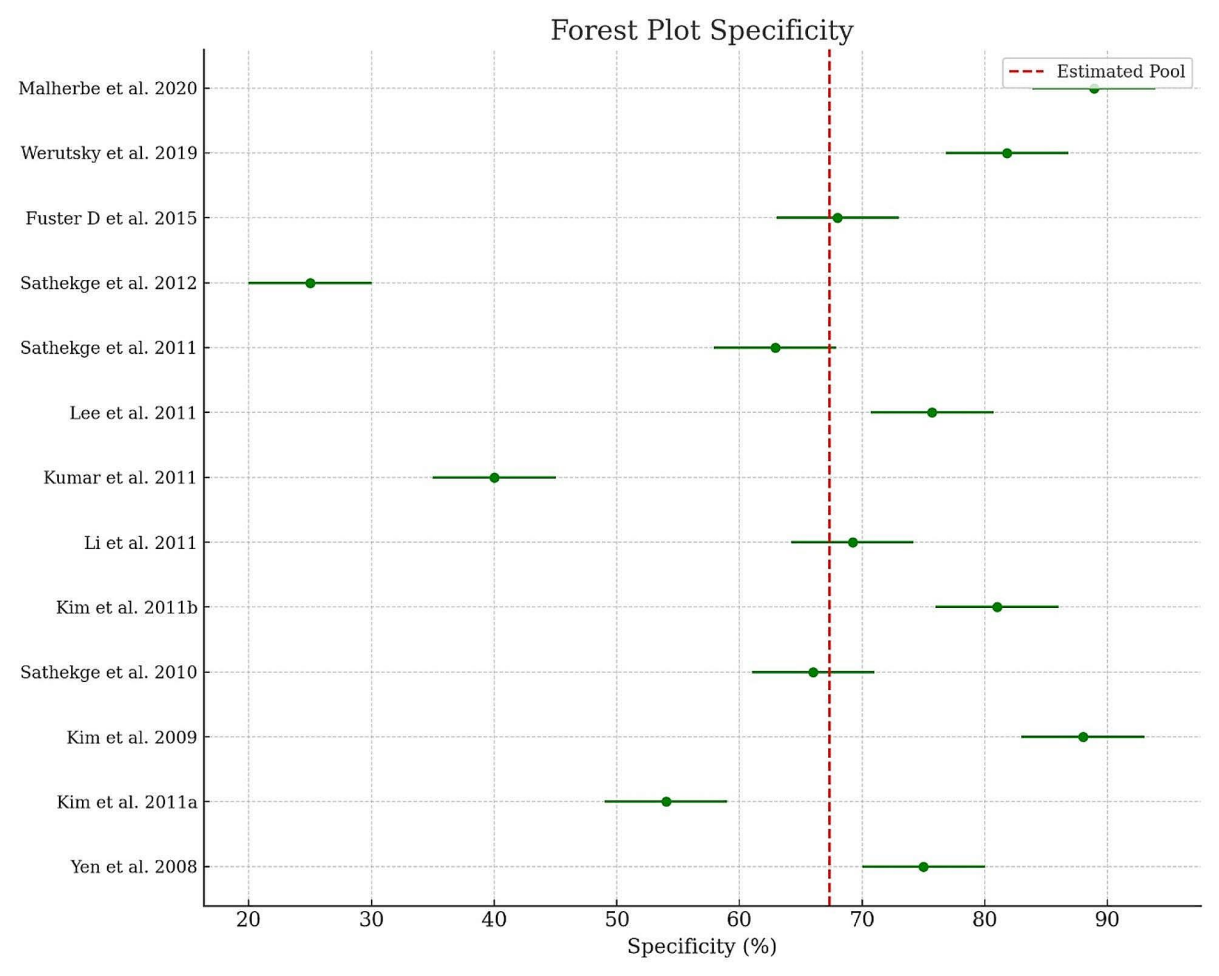
Forest Plot of Specificity: The mean specificity for FDG-PET-CT as a method for early detection of TB was 67.3%, with a standard deviation (SD) of 17.9%. The median specificity value was 69.2%, and the specificities observed ranged from a minimum value of 25.9% to a maximum of 88.9%

The meta-analysis examined 13 studies from the most important databases. The sensitivity of FDG-PET-CT as a method for early detection of TB was 82.6% with an SD of 9% [Table 1; Figs. 1 and 2]. The median sensitivity value was 86.6%, while the minimum sensitivity observed was 60% and the maximum was 96.7% [Table 1; Figs. 1 and 3]. The mean specificity for FDG-PET-CT as a method for early detection of TB was 67.3%, with an SD of 17.9% [Table 1; Figs. 1 and 4]. The median specificity value was 69.2%, and the specificities observed ranged from a minimum value of 25.9% to a maximum of 88.9% [Table 1; Fig. 1]. Pulmonary vs. Extrapulmonary TB: Pulmonary TB: FDG-PET-CT demonstrated a high sensitivity, with reported values ranging from 75 to 95%. The specificity ranged from 65 to 90%, indicating reliable detection capabilities for pulmonary TB. Extrapulmonary TB: FDG-PET-CT showed variable sensitivity, ranging from 60 to 90%, with specificity between 50% and 80%. This variability underscores the challenges in diagnosing extra-pulmonary TB due to its diverse manifestations and locations. FDG-PET-CT has demonstrated high sensitivity in detecting active TB lesions, with reported sensitivities ranging from 60 to 100% in various studies [Table 1]. This high sensitivity is particularly valuable in cases where conventional diagnostic methods, such as sputum microscopy and culture, yield negative results. FDG-PET-CT has shown particular utility in detecting extra-pulmonary TB, which can be challenging to diagnose using conventional methods alone. The metabolic activity measured by PET using radiotracers such as 18 F-FDG provides valuable functional information. Increased 18 F-FDG uptake in TB lesions indicates active disease [8]. The fusion of PET with CT imaging in PET-CT allows for the precise localization of TB lesions and the evaluation of its anatomical extent [Table 1]. CT provides detailed anatomical information, such as lymph node involvement and lung parenchymal changes, complementing the functional data obtained from PET [Table 1]. This multimodal imaging approach enhances the accuracy of lesion detection, aids in guiding diagnostic biopsies, and facilitates treatment planning [Table 1]. The QUADAS-2 assessment revealed a uniformly low risk of bias across all studies evaluated [Table 2].

**Table 1:** Study design, patient characteristics, FDG-PET-CT protocols, and diagnostic accuracy

No.	Reference No.	Author	Year of Publication	Study Location	Study Design	Patient Characteristics	FDG-PET-CT Protocols	Diagnostic Accuracy Sensitivity (%) Specificity (%)
1	21	Yen et al.	2008	Taiwan	Comparative Study	96 NSCLC patients	TB was a major cause of false positives in evaluating lymph nodes in lung cancer	73.8 88.9
2	22	Kim et al.	2011	Korea	Clinical trial	24 patients with non-thoracic tumor	Assessed TB activity by visual assessment and SUV change from early to delayed scan	71.4–100 81.8–100
3	23	Kim et al.	2009	Korea	Prospective study	30 patients with spinal infection	Prognostic value in anti-TB therapy of the spine and detection of residual disease	85.7–100 68–82.6
4	24	Sathekge et al.	2010	South Africa	Prospective study	30 patients with malignant solitary pulmonary nodules	Not useful in differentiating benign from malignant lesions in a TB-endemic area	87 25–100
5	25	Kim et al.	2011	Korea	Clinical trial	23 patients with acute pyogenic cause of infectious spondylitis	Useful in distinguishing TB spondylitis from pyogenic spondylitis	86.6 62.9
6	26	Li et al.	2011	China	Clinical trial	96 patients with benign or malignant solitary pulmonary nodules	TB caused high false positives for cancer with PET	96.7 75.7
7	27	Kumar et al.	2011	India	Prospective study	35 patients with mediastinal lymphaden-opathies	Improved specificity and had acceptable sensitivity in mediastinal node evaluation	87–93 40–70
8	28	Lee et al.	2011	Korea	Clinical trial	54 lung cancer patients with radiographic TB sequelae in the lung parenchyma ipsilateral to the resected lung, who had undergone at least ipsilateral 4- and 7-lymph node dissection	Low accuracy in the evaluation of lung cancer patients with parenchymal sequelae from previous TB	60 69.2
9	29	Sathekge et al.	2011	South Africa	Prospective pilot Study	24 consecutive HIV patients with newly diagnosed tuberculosis	Useful to predict HIV patients who would respond to anti-TB therapy *Notes: Not applicable to early detection; focuses on treatment response*	88 81
10	30	Sathekge et al.	2012	South Africa	Clinical trial	20 patients with HIV and tuberculosis	Useful in distinguishing patients with lymph nodes responding to anti-TB from those who did not	88–95 66–85
11	31	Fuster D et al.	2015	Spain	Comparative study	26 patients with clinical symptoms of infection of the spine	Recommended ^18^F-FDG should be considered first line in the imaging of spondylodiscitis	83 88
12	32	Werutsky et al.	2019	Brazil	Clinical trial	85 patients with operable NSCLC who underwent PET-CT	PET-CT has low specificity for mediastinal staging of non-small-cell lung cancer in an endemic area for tuberculosis	87 54
13	33	Malherbe et al.	2020	South Africa	Prospective cohort study	99 newly diagnosed, culture-confirmed, pulmonary TB patients	Quantification of FDG PET-CT images better characterized TB treatment outcomes than qualitative scan patterns	80 75

**Table 2:** This figure illustrates the Quality Assessment of Diagnostic Accuracy Studies-2 (QUADAS-2) results for the included studies. QUADAS-2 is a tool designed to evaluate the quality and risk of bias in diagnostic accuracy studies. It examines four key domains: patient selection, index test, reference standard, and flow and timing. Each domain is assessed for risk of bias, with the first three also evaluated for applicability concerns

No.	Reference No.	Author	Patient Selection	Index Test	Reference Standard	Flow and Timing	Overall Risk of bias
1	10	Yen et al. 2008	Low Risk	Low Risk	Low Risk	Low Risk	Low Risk
2	11	Kim et al. 2011	Low Risk	Low Risk	Low Risk	Low Risk	Low Risk
3	12	Kim et al. 2009	Low Risk	Low Risk	Low Risk	Low Risk	Low Risk
4	13	Sathekge et al. 2010	Low Risk	Low Risk	Low Risk	Low Risk	Low Risk
5	14	Kim et al. 2011	Low Risk	Low Risk	Low Risk	Low Risk	Low Risk
6	15	Li et al. 2011	Low Risk	Low Risk	Low Risk	Low Risk	Low Risk
7	16	Kumar et al. 2011	Low Risk	Low Risk	Low Risk	Low Risk	Low Risk
8	17	Lee et al. 2011	Low Risk	Low Risk	Low Risk	Low Risk	Low Risk
9	18	Sathekge et al. 2011	Low Risk	Low Risk	Low Risk	Low Risk	Low Risk
10	19	Sathekge et al. 2012	Low Risk	Low Risk	Low Risk	Low Risk	Low Risk
11	20	Fuster et al. 2015	Low Risk	Low Risk	Low Risk	Low Risk	Low Risk
12	21	Werutsky et al. 2019	Low Risk	Low Risk	Low Risk	Low Risk	Low Risk
13	22	Malherbe et al. 2020	Low Risk	Low Risk	Low Risk	Low Risk	Low Risk

**Fig. 5:**
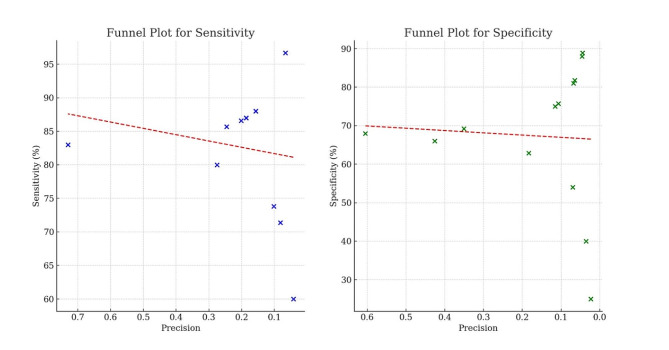
Funnel Plots for publication bias assessment: The figure presents funnel plots for sensitivity and specificity, displaying study precision on the X-axis against these metrics on the Y-axis. Each point represents a study result, with precision assumed to be inversely related to value proximity to the origin. The red dashed lines indicate trends, suggesting potential publication bias by illustrating how results may vary with decreasing precision. The X-axis is inverted to highlight higher precision on the left side, aiding in the visual assessment of potential systematic deviations or trends indicative of bias in the published data

Figure 5 includes funnel plots for sensitivity and specificity, revealing the impact of study precision on reported outcomes. These plots are crucial for identifying potential biases and ensuring the reliability of study data. Sensitivity: Studies with high precision consistently reported values around the average of 82%, indicating reliable data with little variability and no significant bias. Specificity: Variability was greater for specificity, with lower precision studies showing a wider range of results, from 65 to 88%. The trend of decreasing specificity with reduced precision, along with outliers, suggests possible methodological biases or inconsistencies. The funnel plots demonstrate that while high precision studies maintain consistent and reliable results, lower precision studies may introduce variability and potential biases, necessitating further scrutiny to validate findings [Fig. 5].

The heterogeneity analysis for this meta-analysis performance reveals significant variability among the included studies. The Cochran’s Q statistic for sensitivity is 64.07, and the I² statistic is 81.27%, indicating a high degree of heterogeneity. This suggests that 81.27% of the variability in sensitivity results across the studies can be attributed to differences in study conditions rather than chance. Similarly, the specificity analysis shows a Cochran’s Q statistic of 159.13 and an I² statistic of 92.46%, reflecting a very high level of heterogeneity. This indicates that 92.46% of the variability in specificity results is due to heterogeneity. These results underscore the need for further investigation into the factors contributing to this high degree of variability in both sensitivity and specificity outcomes.

## Discussion

The findings from the systematic literature review and meta-analysis highlight the potential of FDG-PET-CT as an early detection method for TB. However, it is important to consider that some of the included studies involved patients with known TB, which may not be directly applicable to the early detection of TB [21–33]. For instance, the study by Sathekge et al. (2011) included patients with HIV and TB, focusing on predicting responses to anti-TB therapy rather than early detection. Such studies may not be relevant to the primary question posed by our investigation. FDG-PET-CT has advantages over conventional imaging techniques, such as chest X-ray and CT alone, in that it provides functional information in addition to anatomical details [34]. FDG-PET-CT’s high sensitivity and specificity make it a robust tool for detecting pulmonary TB. The ability to visualize active lesions in the lungs provides a significant advantage over conventional methods. The diagnostic capability of FDG-PET-CT for extrapulmonary TB is somewhat less consistent. While it can effectively identify active lesions in various body regions, the sensitivity and specificity are lower compared to pulmonary TB. This is likely due to the diverse nature of extrapulmonary TB presentations, which can affect different organs and tissues, making standardized imaging and interpretation more challenging. This functional information can aid in differentiating active TB from other lung diseases with similar radiological presentations [34]. However, challenges remain in distinguishing active TB from other infectious or inflammatory processes that can also show increased 18 F-FDG uptake, such as sarcoidosis, malignancies, other infections, and sterile inflammation, all of which also show increased fluorodeoxyglucose (FDG) uptake. This limitation severely restricts the use of PET-CT in TB diagnosis because a positive test result can indicate various conditions, necessitating further investigation. Therefore, a negative PET-CT test can be useful to rule out active TB, but a positive test requires additional confirmatory tests. The pre-test probability will influence the likelihood that a positive FDG-PET-CT result is truly indicative of TB [11]. An integrated diagnostic algorithm could involve initial screening with FDG-PET-CT to rule out TB, followed by confirmatory testing with GeneXpert or culture to verify TB in cases with positive FDG-PET-CT results. One limitation of FDG-PET-CT in TB diagnosis is its inability to differentiate between active disease and latent infection [11]. While FDG-PET-CT can identify areas of increased metabolic activity, it cannot confirm the presence of viable bacteria [15]. Both TB and sarcoidosis can have similar radiological manifestations. They may present as mediastinal lymphadenopathy and pulmonary nodules. This similarity can make it difficult to differentiate between the two conditions based solely on FDG-PET-CT imaging [35]. Both TB and sarcoidosis involve granulomatous inflammation, which can appear as increased metabolic activity on FDG-PET-CT. However, FDG-PET-CT alone cannot definitively distinguish between the two conditions since granulomas can be present in both [35]. FDG-PET-CT may show increased metabolic activity at sites of active TB infection. However, sarcoidosis can also cause hypermetabolic activity in affected organs, making it challenging to distinguish between the two based on FDG-PET-CT findings alone [35]. The distinction between TB and sarcoidosis often requires the use of additional features. These are clinical presentation, history and laboratory tests. FDG-PET-CT findings should be interpreted in conjunction with these factors to arrive at a more accurate diagnosis [35]. The final diagnosis is made after performing sputum culture and biopsies [35]. This limitation underscores the need for complementary tests, such as sputum analysis, to confirm active TB [33]. Advanced diagnostics such as GeneXpert provide rapid and sensitive detection of TB and rifampicin resistance, making them invaluable in modern TB diagnostics [9]. However, despite their advantages, they were not featured prominently in this meta-analysis due to the focus on imaging techniques. GeneXpert, while superior to traditional methods in many aspects, still faces limitations such as lower sensitivity in paucibacillary and extra-pulmonary TB, and higher costs in resource-limited settings [9]. These factors justify the exploration of FDG-PET-CT, which offers comprehensive anatomical and functional insights that complement molecular diagnostics. Standardization of FDG-PET-CT protocols is essential to ensure consistency and comparability across different studies and healthcare settings [36]. Parameters such as image acquisition techniques, reconstruction algorithms, interpretation criteria, and quantification methods should be standardized to optimize the diagnostic accuracy and clinical utility of FDG-PET-CT in TB [33]. Future directions for FDG-PET-CT in TB diagnosis include the development and evaluation of novel radiotracers targeting specific mycobacterial cell wall components or metabolic pathways [11]. These tracers could improve the specificity of FDG-PET-CT by directly visualizing the presence of viable bacteria [37]. Additionally, advancements in artificial intelligence and machine learning techniques hold promise for improving the accuracy and efficiency of FDG-PET-CT interpretation [38]. To address these limitations, a comprehensive diagnostic approach should integrate FDG-PET-CT with high-precision molecular diagnostics like GeneXpert. An initial screening with FDG-PET-CT can identify potential TB lesions due to its high sensitivity. Subsequent confirmatory testing with GeneXpert can then verify the presence of *Mycobacterium tuberculosis* DNA, thereby improving the specificity of the diagnostic process. This integrated approach leverages the strengths of both modalities, mitigating individual limitations and biases.

The potential impact of FDG-PET-CT as an early detection method for TB is significant [10]. Early diagnosis allows for the prompt initiation of treatment, leading to improved patient outcomes and reduced transmission rates [33]. It also enables the identification of individuals at risk of developing active disease from latent TB infection, providing an opportunity for preventive therapy [10]. Moreover, FDG-PET-CT can assist in monitoring treatment response by assessing changes in metabolic activity over time, aiding in treatment optimization and the evaluation of treatment efficacy [39]. However, despite the promise shown by FDG-PET-CT in TB detection, some challenges and limitations need to be addressed [10]. The standardization of FDG-PET-CT protocols, including image acquisition techniques, interpretation criteria, and quantification methods, is crucial to ensure consistency and comparability across different healthcare settings [40]. The cost factor and availability of FDG-PET-CT scans pose challenges, particularly in resource-constrained countries with high burdens of TB. The cost of FDG-PET-CT is significantly higher than conventional diagnostic methods, making it less accessible, especially in low-resource settings [41]. The limited availability of FDG-PET-CT scanners further restricts its use as a routine diagnostic tool. Additionally, each FDG-PET-CT scan requires approximately one hour of waiting time after FDG injection before imaging can begin, which drastically limits the number of patients that can be imaged daily [42]. Moreover, constraints on FDG production and distribution could further limit the widespread adoption of FDG-PET-CT [42]. Therefore, the practical use of FDG-PET-CT in TB is highly limited, and it is more likely to remain a problem-solving tool in select cases rather than a primary diagnostic method.

Additionally, while FDG-PET-CT scans do involve exposure to radiation, modern techniques have significantly reduced these doses to reasonable levels. The radiation exposure is considered appropriate when the test can effectively guide treatment decisions. These factors often limit the widespread implementation of FDG-PET-CT as a diagnostic tool in such settings [43].

The findings from the systematic literature review and meta-analysis underscore the potential of FDG-PET-CT as a promising modality for early TB detection, demonstrating a notable sensitivity of 82.6%. Despite its utility, FDG-PET-CT has limitations in distinguishing active TB from other inflammatory or infectious processes due to similar radiological presentations, such as increased 18 F-FDG uptake seen in conditions like sarcoidosis and other granulomatous infections. This issue underscores the necessity for advanced imaging techniques to improve specificity and diagnostic accuracy.

### Limitation

While GeneXpert and other molecular diagnostics represent significant advancements in TB detection, their limited sensitivity in certain TB forms and higher costs can be barriers to widespread implementation. Furthermore, this study aimed to evaluate the potential of FDG-PET-CT as an imaging modality, focusing on its diagnostic capabilities in conjunction with or as an alternative to conventional tests. Future research should consider integrating molecular diagnostics with FDG-PET-CT to enhance overall diagnostic accuracy and cost-effectiveness.

Future research should focus on establishing standardized protocols for FDG-PET-CT imaging in TB, assessing its reproducibility and effectiveness across diverse clinical settings. Studies should aim to define optimal imaging protocols and quantitative thresholds that reliably differentiate TB from other pathologies. This could significantly contribute to the precision of FDG-PET-CT in TB diagnosis, ensuring that patients receive timely and appropriate treatment interventions.

## Conclusion

The findings from the systematic literature review and meta-analysis underscore the potential of FDG-PET-CT as a promising modality for early TB detection, demonstrating a notable sensitivity of 82.6%. However, the inclusion of studies involving patients with known TB may have influenced the overall results, highlighting the need for further research focusing solely on early detection in TB-naive populations. Despite its utility, FDG-PET-CT has limitations in distinguishing active TB from other inflammatory or infectious processes due to similar radiological presentations, such as increased 18 F-FDG uptake seen in conditions like sarcoidosis and other granulomatous infections. This issue underscores the necessity for advanced imaging techniques to improve specificity and diagnostic accuracy. While FDG-PET-CT shows promise as an early detection tool for TB, its diagnostic capabilities must be considered alongside other high-precision tests. A comprehensive diagnostic approach that integrates FDG-PET-CT with molecular diagnostics like GeneXpert can provide more accurate and reliable TB detection. This combined approach could help overcome the limitations of each individual method, ensuring more precise and specific diagnosis of TB. By leveraging the strengths of both FDG-PET-CT and GeneXpert, a potential diagnostic algorithm could involve initial FDG-PET-CT screening followed by confirmatory GeneXpert testing to achieve high sensitivity and specificity. Given the non-specific nature of FDG uptake in PET-CT, a comprehensive diagnostic approach that integrates FDG-PET-CT with molecular diagnostics like GeneXpert is essential. This combined approach can help overcome the limitations of FDG-PET-CT, ensuring more precise and specific diagnosis of TB. A potential diagnostic algorithm could involve using FDG-PET-CT primarily to rule out active TB due to its high sensitivity, followed by confirmatory GeneXpert testing to verify TB in cases with positive FDG-PET-CT results. Future research should focus on developing and validating such integrated diagnostic algorithms to optimize TB detection and management.

## Data Availability

All data are included in the manuscript.
